# Cuproptosis-related modification patterns depict the tumor microenvironment, precision immunotherapy, and prognosis of kidney renal clear cell carcinoma

**DOI:** 10.3389/fimmu.2022.933241

**Published:** 2022-09-23

**Authors:** Zhiyong Cai, You'e He, Zhengzheng Yu, Jiao Hu, Zicheng Xiao, Xiongbing Zu, Zhenghao Li, Huihuang Li

**Affiliations:** ^1^ Department of Urology, Xiangya Hospital, Central South University, Changsha, China; ^2^ National Clinical Research Center for Geriatric Disorder, Xiangya Hospital, Central South University, Changsha, China; ^3^ Research Center of Carcinogenesis and Targeted Therapy, Xiangya Hospital, Central South University, Changsha, China; ^4^ Hunan Provincial Key Laboratory of Hepatobiliary Disease Research and Division of Hepato-Biliary-Pancreatic Surgery, Department of General Surgery, The Second Xiangya Hospital, Central South University, Changsha, China

**Keywords:** cuproptosis, KIRC, tumor microenvironment, immunotherapy, prognosis

## Abstract

**Background:**

Due to the different infiltration abundance of immune cells in tumor, the efficacy of immunotherapy varies widely among individuals. Recently, growing evidence suggested that cuproptosis has impact on cancer immunity profoundly. However, the comprehensive roles of cuproptosis-related genes in tumor microenvironment (TME) and in response to immunotherapy are still unclear.

**Methods:**

Based on 43 cuproptosis-related genes, we employed unsupervised clustering to identify cuproptosis-related patterns and single-sample gene set enrichment analysis algorithm to build a cuproptosis signature for individual patient’s immune cell infiltration and efficacy of immune checkpoint blockade (ICB) evaluation. Then, the cuproptosis-related genes were narrowed down using univariate Cox regression model and least absolute shrinkage and selection operator algorithm. Finally, a cuproptosis risk score was built by random survival forest based on these narrowed-down genes.

**Results:**

Two distinct cuproptosis-related patterns were developed, with cuproptosis cluster 1 showing better prognosis and higher enrichment of immune-related pathways and infiltration of immune cells. For individual evaluation, the cuproptosis signature that we built could be used not only for predicting immune cell infiltration in TME but also for evaluating an individual’s sensitivity to ICBs. Patients with higher cuproptosis signature scores exhibited more activated cancer immune processes, higher immune cell infiltration, and better curative efficacy of ICBs. Furthermore, a robust cuproptosis risk score indicated that patients with higher risk scores showed worse survival outcomes, which could be validated in internal and external validation cohorts. Ultimately, a nomogram which combined the risk score with the prognostic clinical factors was developed, and it showed excellent prediction accuracy for survival outcomes.

**Conclusion:**

Distinct cuproptosis-related patterns have significant differences on prognosis and immune cell infiltration in kidney renal clear cell carcinoma (KIRC). Cuproptosis signature and risk score are able to provide guidance for precision therapy and accurate prognosis prediction for patients with KIRC.

## Introduction

Kidney renal clear cell carcinoma (KIRC) is the most common malignant tumor in renal cell carcinoma (RCC) (1). It is estimated that over 75,000 cases occur and that 13,000 patients die each year (2). Although the prognosis of localized KIRC is favorable, the 5-year overall survival (OS) rate is lower than 10% in metastatic KIRC (mKIRC) (3). Recently, immunotherapy, especially immune checkpoint inhibitors (ICB), has achieved brilliant efficacy in mKIRC individuals (4). The CheckMate 214 clinical trial has reported that intermediate-risk and poor-risk patients can benefit more from ICB treatment than targeted therapy (5). However, only a proportion of patients can produce an anti-tumor response (6). Hence, it is urgent to find a predictor which can guide clinicians for ICB application.

The tumor microenvironment (TME) is a complicated tissue environment, which consists of various immune cells, stromal cells, and noncellular components (7). Hence, there is a large heterogeneity in the TME. According to the infiltration levels of tumor-fighting effector cells and inflammatory cytokines, the TME can be sorted into inflamed type and non-inflamed type in brief (8). Meanwhile, several large-scale studies have demonstrated that the abundance of pre-existing infiltrated immune cells determines the efficacy of ICB therapy (9–12).

Cuproptosis, a brand-new concept which is defined as copper-dependent cell death, is accompanied with elevated mitochondrial-dependent energy metabolism and accumulation of reactive oxygen species (ROS) (13). Intriguingly, several studies reported that cuproptosis-related genes, such as ATOX1 and CP, can affect the progression of cancer (14, 15). More importantly, Voli et al. found that the variation of copper transporter 1 has influence on the expression of programmed cell death 1 ligand 1 (PD-L1) and the infiltration quantity of CD8^+^T cells and NK cells in the TME (16). However, all these studies merely focused on one or two cuproptosis-related genes and their roles in the TME. A comprehensive analysis of multiple cuproptosis-related genes and their roles in assessing the TME, efficacy of ICB, and prognosis in KIRC is lacking. So, we systematically correlated cuproptosis-related genes with the TME as well as their sensitivity to immunotherapy in KIRC for the first time.

## Methods

### Data collection and processing

The high-throughput sequencing data of mRNA, clinically associated data, and survival data of The Cancer Genome Atlas (TCGA)-KIRC (526 samples) were downloaded from UCSC Xena (http://xena.ucsc.edu/) (17). The expression matrix of mRNA was transformed from fragments per kilobase per million mapped fragments to transcripts per kilobase million. As an external validation set, the E-MTAB-1980 KIRC cohort (101 samples) was downloaded from ArrayExpress (https://www.ebi.ac.uk/arrayexpress/). Furthermore, as our previous study has reported (18), 46 renal cell carcinoma samples were collected from our hospital and named the Xiangya-RCC cohort.

Immunotherapy cohorts were downloaded from the study of Gide et al. (PMID30753825 cohort, anti-PD-1 or combined anti-PD-1 with anti-CTLA-4) (19), the study of Kim et al. (PMID30013197, anti-PD-1/PD-L1) (20), the study of Allen et al. (PMID26359337, anti-CTLA-4) (21), GSE35640 (MAGE-A3 immunotherapy), GSE111636 (anti-PD-1 immunotherapy), GSE126044 (anti-PD-1 treatment), GSE173839 (anti-PD-L1 treatment), and GSE135222 (anti-PD-1/PD-L1 treatment).

### Unsupervised clustering for cuproptosis-related patterns

Forty-three cuproptosis-related genes were selected from the review of Chang et al. (13) (**Supplementary Table S1**). Consensus clustering algorithm (maxK = 5, reps = 1,000, pItem = 0.8, distance = “manhattan”, clusterAIg = “pam”) was employed to judge the quantity and stability of clusters based on these genes (ConsensuClusterPlus R package) (22).

### Differentially expressed genes and functional analysis

Limma R package was used to screen differentially expressed genes (DEGs) between cuproptosis-related patterns, and the screen criteria were set as |log fold change| >1 and adjusted *p*-value <0.05.

Gene Ontology (GO) and Kyoto Encyclopedia of Genes and Genomes (KEGG) analysis were conducted using Gene Set Enrichment Analysis (GSEA) algorithm in GSVA R package. The gmt files “c2.cp.kegg.v6.2.symbols.gmt” and “h.all.v7.2.symbols.gmt” were downloaded from the molecular signature database (http://www.gsea-msigdb.org/gsea/msigdb). |Normalized enrichment score| >1 and adjusted *p*-value <0.05 were used as criteria for significant enrichment.

### Description of the tumor immune characteristics of KIRC and construction of the cuproptosis signature

We described the TME of KIRC in five aspects: Firstly, seven steps of cancer immunity cycle were analyzed by using the Tracking Tumor Immunophenotype website (http://biocc.hrbmu.edu.cn/TIP/) (23). Secondly, cell markers of tumor-infiltrating leukocytes (TILs) were downloaded from the study of Charoentong (24), and infiltration of TILs was calculated using single-sample gene set enrichment analysis (ssGSEA) algorithm. A cuproptosis signature was also generated using ssGSEA based on 43 cuproptosis-related genes. In order to eliminate the effects of different algorithms, another three algorithms for immune cell calculation, including MCP, Quantiseq, and TIMER, were also applied. Thirdly, the effector genes of immune cells were gathered from our previous study (25). Fourthly, 22 inhibitory immune checkpoints were assembled from the study of Auslander et al. (26). Finally, T cell inflamed score (TIS) was employed to evaluate the potential response probability to ICB (25).

### Analysis of scRNA-seq cohort

Seven single-cell RNA sequencing (scRNA-seq) count matrixes of KIRC were downloaded from the supplemental material of GSE159115 (27). We then converted the seven matrixes into Seurat objects using the CreateSeuratObject function (Seurat R package, version 4.1.1). Single cells with less than 1,000 UMIs or less than 200 genes or with a value of log_10_ genes per UMI less than 0.70 or more than 20% mitochondrion-derived UMI counts were regarded as low-quality cells and filtered out for further analysis. Based on the top 3,000 variable genes, we then integrated seven samples into one Seurat object using the IntegrateData function in Seurat to eliminate batch effects. We then identified the main cell clusters using the FindClusters function in Seurat (resolution = 0.4) and visualized these cell clusters using uniform manifold approximation and projection. The cell clusters were first recognized using SingleR R package, and then the cell types were confirmed based on the markers obtained from previous studies (27, 28).

### Construction and validation of the cuproptosis risk score

A total of 43 cuproptosis-related genes were used to screen genes possessing univariate prognostic values by univariate Cox analysis. These prognostic genes were further narrowed down by least absolute shrinkage and selection operator (LASSO) regression with minimal lambda (0.05). Subsequently, a cuproptosis risk score was constructed using the “rfsrc” function in “randomForestSRC” R package based on the expression of these genes.

Individuals in the TCGA-KIRC cohort were randomly divided into training and internal validation cohorts in a 7:3 ratio, while the E-MTAB-1980 KIRC cohort was set as the external validation cohort. According to the median value of risk score, we classified individuals into high-risk group and low-risk group. Kaplan–Meier (K–M) survival curve and log-rank test were employed to compare the prognosis difference between two groups by survminer R package. In additional, tROC R package was used to estimate the prediction reliability of risk score.

### Establishment of a nomogram

Univariate Cox analyses, along with multivariate Cox analyses, were employed to filter the independent impact factor in cohorts. Then, rms R package was applied to establish the nomogram. Subsequently, calibration curves and time-dependent receiver operating characteristic curves were used to estimate the clinical relevance and prediction accuracy, respectively.

### Statistical analysis

*T*-test or Mann–Whitney *U*-test was employed to compare the continuous variables between two groups. Chi-square test or fisher exact test was used to compare differences between groups with dichotomous variables. Pearson or Spearman correlation analysis was conducted to assess the relation between different factors. *P <*0.05 was set as criterion for judging a significant difference. Two-side statistical tests were used. R software (version 4.1.3) was applied throughout the analysis.

## Results

### Development of cuproptosis-related patterns

**Figure 1:**
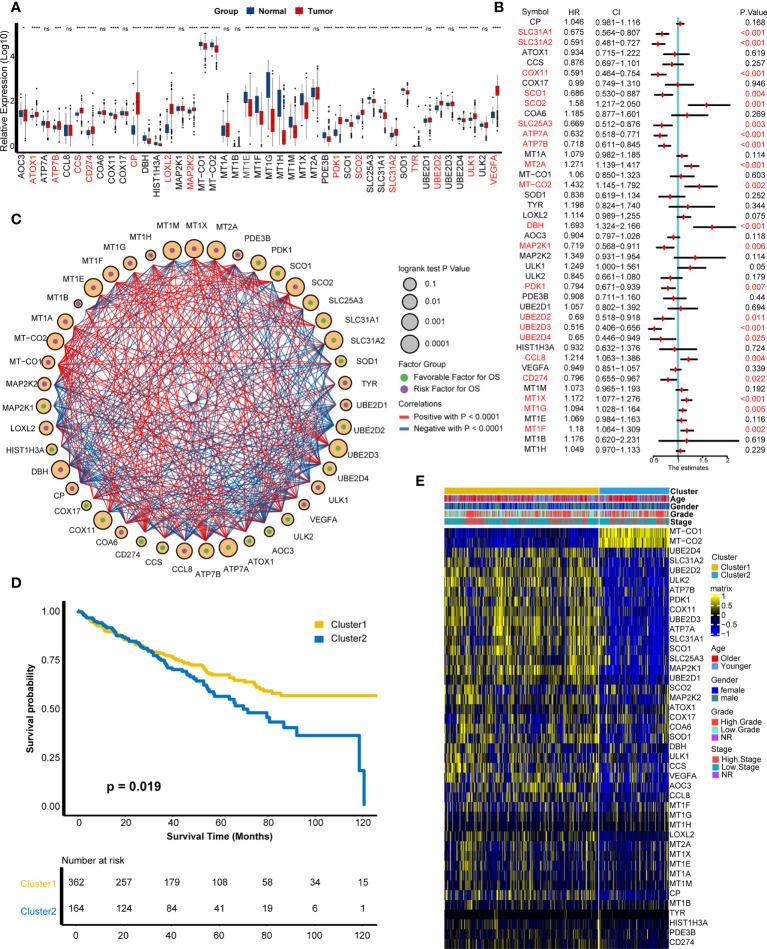
Development of cuproptosis-related patterns. **(A)** Expression of cuproptosis-related genes in tumors and adjacent normal tissues. **(B)** Prognostic analysis of cuproptosis-related genes using univariate Cox regression. **(C)** Correlation analysis among cuproptosis-related genes. The size of the circle represented the *p*-value of overall survival, the green and purple dots in the circle meant favorable and risk factor in prognosis, and the red and blue lines that connected two circles meant positive and negative regulating relationships, respectively. **(D)** Survival outcome between two patterns. **(E)** Distribution of clinical features (age, gender, grade, and stage) and expression matrix of cuproptosis-related patterns. *p < 0.05, ***p < 0.001, ****p < 0.0001; ns, not statistically significant.

On the basis of the 43 cuproptosis-related genes, their mRNA expression levels were compared between tumor tissues and adjacent normal tissues in the TCGA-KIRC cohort (**Figure 1A**). We found that 32 genes showed significant differences on RNA expression, containing ATOX1, ATP7B, CCS, CD274, CP, LOXL2, MAP2K2, PDK1, SCO2, SLC31A2, TYR, UBE2D2, ULK1, VEGFA, and so on. Next, a univariate Cox analysis was employed to filter cuproptosis-related genes with prognostic value and found 21 genes with a significant prognostic value (**Figure 1B**, **Supplementary Figure S1**, **Supplementary Table S2**), including SCO2, MT2A, DBH, CCL8, MT1G, MT1X, MT-CO2, MT1F and so on. To explore the interaction probability among cuproptosis-related genes, a comprehensive correlation analysis was implemented and found intimate interactions among them (**Figure 1C**). Therefore, an unsupervised clustering analysis was conducted based on cuproptosis-related genes and found two distinct clusters (**Supplementary Figure S2**), which were named as cuproptosis-related patterns. Furthermore, 362 individuals and 164 individuals were divided into cluster 1 and cluster 2, respectively. As shown in **Figure 1D**, cluster 1 has a better survival outcome than cluster 2 (*p* = 0.019). The distribution of clinical features (age, gender, tumor grade, and tumor stage) and the distinct expression modes between two patterns were displayed in a heat map (**Figure 1E**).

### Expression patterns of cuproptosis-related genes on the single-cell level

**Figure 2:**
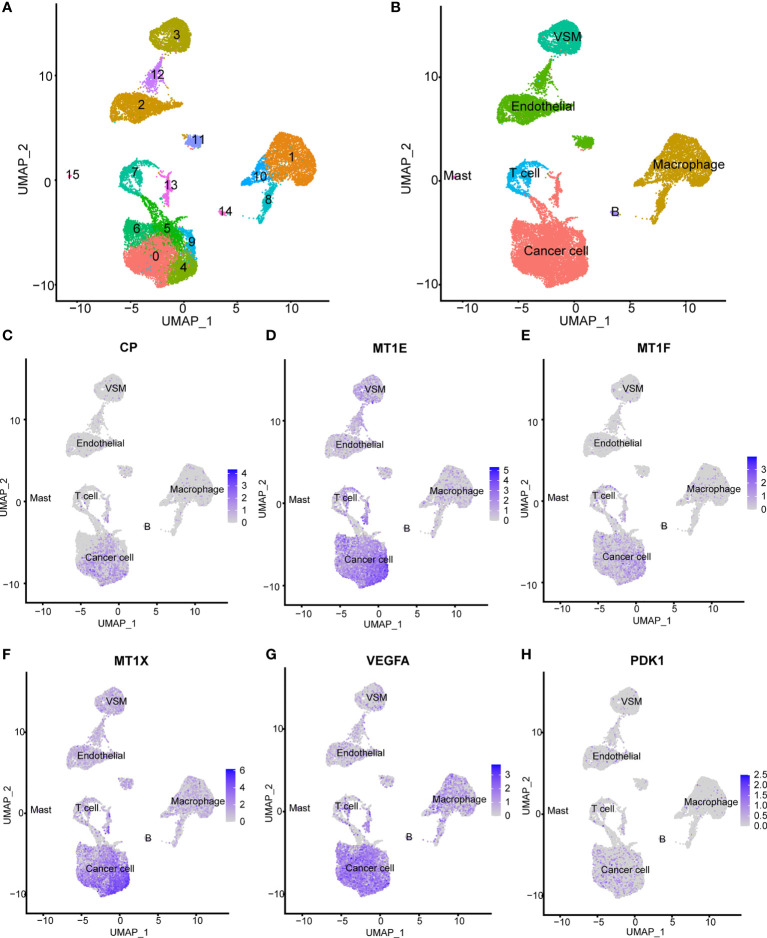
Expression patterns of cuproptosis-related genes on the single-cell level. **(A)** Fifteen main cell clusters in the scRNA KIRC cohort. **(B)** Six recognized cell types based on previous cell markers: cancer cells, endothelial cells, vascular smooth muscle cells, T cells, B cells, and macrophage cells. **(C–H)** Selected cuproptosis-related genes were expressed significantly higher in cancer cells: CP, MT1E, MT1F, MT1X, VEGFA, and PDK1.

Seven samples from seven KIRC patients were involved in this analysis. After quality control, a total of 20,900 single cells were included for further analysis. As shown in **Figure 2A**, these cells could be classified into 15 main cell clusters. Then, these cell clusters were recognized based on the cell markers reported in previous studies: cancer cells (“NDUFA4L2”, “CA9”, “SLC17A3”, and “NNMT”) (**Supplementary Figure S3A**), endothelial cells (“KDR”, “PECAM1”, “ESM1”, and “PLVAP”) (**Supplementary Figure S3B**), vascular smooth muscle (VSM) cells (“ACTA2”, “PDGFRB”, “CNN1”, and “MYH11”) (**Supplementary Figure S3C**), macrophage cells (“LYZ”, “CD68”, “CD163”, and “HLA-DRA”) (**Supplementary Figure S3D**), T cells (“CD3D”, “CD3E”, and “CD3G”) (**Supplementary Figure S3E**), B cells (“CD79A”) (**Supplementary Figure S3F**), and mast cells (“TPSAB1”, “CPA3”, and “MS4A2”) (**Supplementary Figure S3G**) (27, 28). In addition to cancer cells, two kinds of normal cell types, including endothelial and VSM cells, and four kinds of immune cell types, including macrophage, T cells, B cells, and mast cells, were identified (**Figure 2B**). We divided these cell types into cancer cells and non-cancer cells and compared the expression patterns of cuproptosis-related genes (**Supplementary Figure S4A**, **Supplementary Table S3**). A majority of cuproptosis-related genes like CP, MT1E, MT1F, MT1X, VEGFA, and PDK1 were expressed significantly higher on cancer cells (**Figures 2C–H**), indicating that cuproptosis might occur mainly on cancer cells. However, the detailed mechanism needs to be further explored *in vivo* and *in vitro*.

### Functional enrichment analysis and cancer immunity assessment of cuproptosis-based patterns

On account of significant difference in prognosis between two patterns, we intended to explore the underlying mechanism. DEGs were displayed in a heat map and a volcano plot (**Supplementary Figures S4B, C**, **Supplementary Table S4**). Surprisingly, there were several immune-related genes, such as ST8SIA6, CNTN1, KCNK2, F13A1, MTRNR2L12, PVALB, MT-ATP8, and KLK3. Friedman et al. reported that the overexpression of ST8SIA6 can change tumor growth by suppressing the immune response (29). Lin et al. demonstrated that a varied expression of KCNK2 can affect the infiltration of immune cells (30). Consequently, these findings inferred that cuproptosis-related patterns link with immune-related pathways.

**Figure 3:**
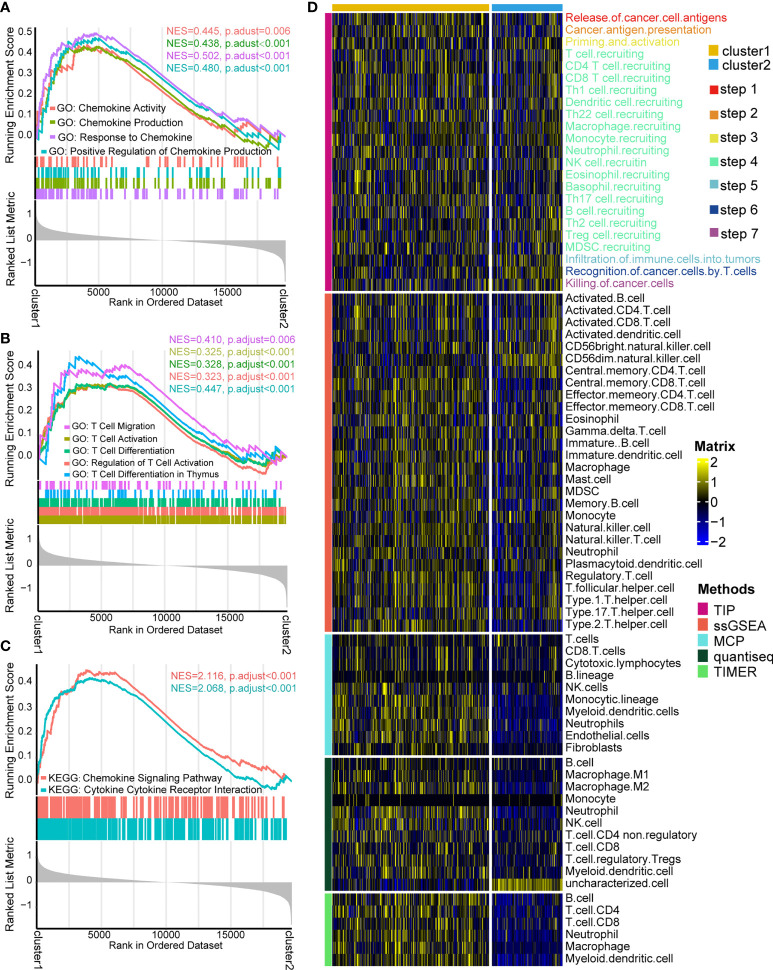
Functional enrichment analysis and cancer immunity assessment of cuproptosis-based patterns. **(A)** Gene Ontology (GO) functional enrichment analysis of chemokine-related pathways. **(B)** GO functional enrichment analysis of T cell-related pathways. **(C)** Kyoto Encyclopedia of Genes and Genomes functional enrichment analysis of cytokine/chemokine-related signaling pathways. **(D)** Expression matrix of cancer immunity cycles and tumor-infiltrating leukocytes between two patterns.

According to the fold change value of all genes in TCGA-KIRC between two patterns, GSEA algorithm was employed to ascertain the detailed enrichment pathways. GO analysis indicated that the pathways of chemokine activity, chemokine production, response to chemokine, and positive regulation of chemokine production were significantly activated in cluster 1 (**Figure 3A**, **Supplementary Table S5**). Furthermore, GO analysis also revealed that the activity of pathways of T cell migration, T cell activation, T cell differentiation, regulation of T cell activation, and T cell differentiation in thymus were enhanced in cluster 1 (**Figure 3B**, **Supplementary Table S5**). KEGG analysis exhibited that the activities of chemokine signaling pathways and cytokine–cytokine receptor interaction signaling pathways were significantly upregulated in cluster 1 (**Figure 3C**, **Supplementary Table S6**). These findings indicated that individuals in cluster 1 had a more vibrant tumor-fighting process than those in cluster 2.

The cancer immunity cycle, containing the initiation of tumor immunity to killing cancer cells by infiltrated T cells (31, 32), acts a critical part in the TME. It was of great interest to us whether there are differences in cancer immunity steps between two patterns. As expected, cluster 1 showed a more active process than cluster 2, including recognition of cancer antigen, initiating response of immune cells, T cell recruiting, and killing cancer cell by TILs (**Figure 3D**). The outcome can infer that cluster 1 probably represents an inflamed type of the TME and produces a better response to immunotherapy (33, 34). Hence, the infiltration levels of various immune cells in the TME were compared between two patterns through four independent algorithms (ssGSEA, MCP, Quantiseq, and TIMER). Consistently, cluster 1 had a higher infiltration level of TILs than cluster 2, containing CD4^+^T cells, CD8^+^T cells, myeloid dendritic cells, macrophage cells, monocytes, B cells, and neutrophils (**Figure 3D**). The result demonstrated that two cuproptosis-related patterns represent two types of the TME: inflamed TME and non-inflamed type.

### Estimating the infiltration level of TILs and the efficacy of ICB on individuals by cuproptosis signature

Although cuproptosis-related patterns played an essential role on distinguishing the infiltration level of TILs in the whole cohort, the patterns lacked the ability to evaluate an individual patient’s TME status. Consequently, we constructed a cuproptosis signature based on these 43 cuproptosis-related genes by ssGSEA algorithm to estimate the abundance of TILs and the efficacy of ICB for individual patients.

**Figure 4:**
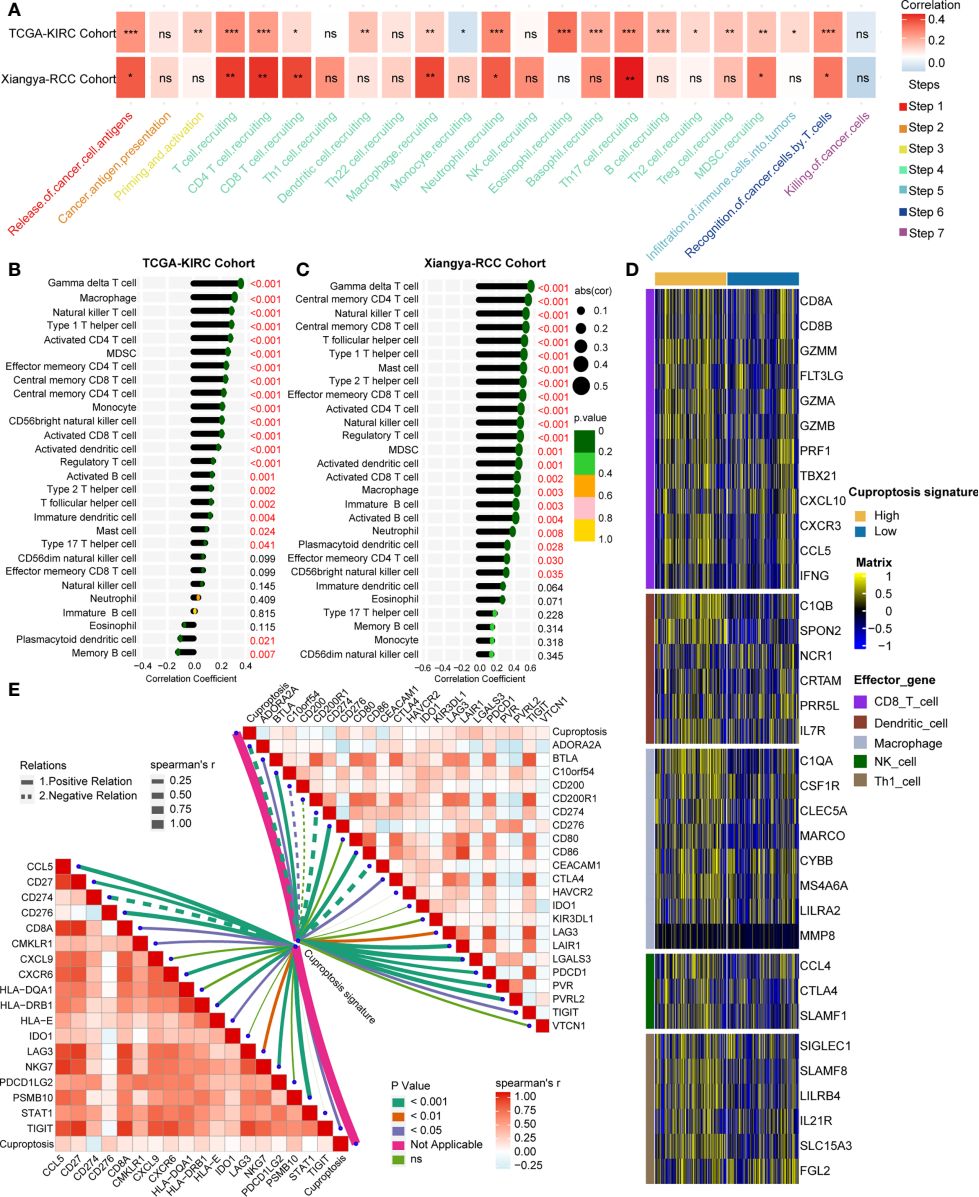
Estimated infiltration level of tumor-infiltrating leukocytes (TILs) and efficacy of immune checkpoint blockade on individuals by cuproptosis signature. **(A)** Correlation analysis on cuproptosis signature and cancer immunity steps in the TCGA-KIRC and Xiangya-RCC cohorts. *p < 0.05, **p < 0.01, ***p < 0.001; ns, not statistically significant. **(B, C)** Correlation analysis on cuproptosis signature and TILs in the TCGA-KIRC and Xiangya-RCC cohorts. **(D)** Expression matrix of TILs (CD8^+^T cell, dendritic cell, macrophage cell, NK cell, and Th1 cell in the high- and low-score signature groups. **(E)** Correlation analysis on cuproptosis signature and T cell inflamed score (left) and inhibitory immune checkpoints (right). The solid and dotted lines represent positive and negative connections, respectively; the thickness of the lines represents the coefficient of the relations; and the diverse colors of the lines represent the *p*-values of the relations.

Interestingly, the cuproptosis signature was significantly positively related to major steps of the cancer immunity cycle both in the TCGA-KIRC cohort and the Xiangya-RCC cohort (**Figure 4A**, **Supplementary Tables S7**, **S8**), including release of tumor antigen, recognition of tumor cell by T cell, and recruitment of diverse immune cells (T cell, macrophage, neutrophil, and Th17). To validate this finding, the cuproptosis signature was directly associated with infiltration of TILs in the TCGA-KIRC cohort and the Xiangya-RCC cohort. In line with a previous outcome, the cuproptosis signature showed a significantly positive connection to infiltration of TILs (**Figures 4B, C**, **Supplementary Tables S9**, **S10**), containing gamma delta T cell, activated CD8^+^ T cell, activated dendritic cell, activated B cell, natural killer T cell, type 1 T helper cell (Th1 cell), type 2 T helper cell (Th2 cell), and so on. Meanwhile, we compared the effector genes of main TILs (CD8^+^T cell, dendritic cell, macrophage cell, NK cell, and Th1 cell) between high- and low-cuproptosis-signature groups. As anticipated, the effector genes of TILs were expressed higher in the high-score signature group (**Figure 4D**). These findings demonstrated that cuproptosis signature is capable of assessing the level of infiltrated immune cells on individuals.

It is widely thought that inflamed TME is the foundation of producing a response to immunotherapy (35, 36). Therefore, TIS, which can predict the efficacy of immunotherapy (37, 38) along with inhibitory immune checkpoints, was further studied. A total of 18 TLS-related genes and 22 ICB-associated genes were collected and were used to correlate with the cuproptosis signature. Collectively, the signature showed significant positive relations with TIS and inhibitory immune checkpoints (**Figure 4E**, **Supplementary Tables S11**, **S12**), from which it can be inferred that the cuproptosis signature has the capacity to predict the efficacy of immunotherapy.

### Direct comparison of ICBs’ efficacy in multiple immunotherapy cohorts

**Figure 5:**
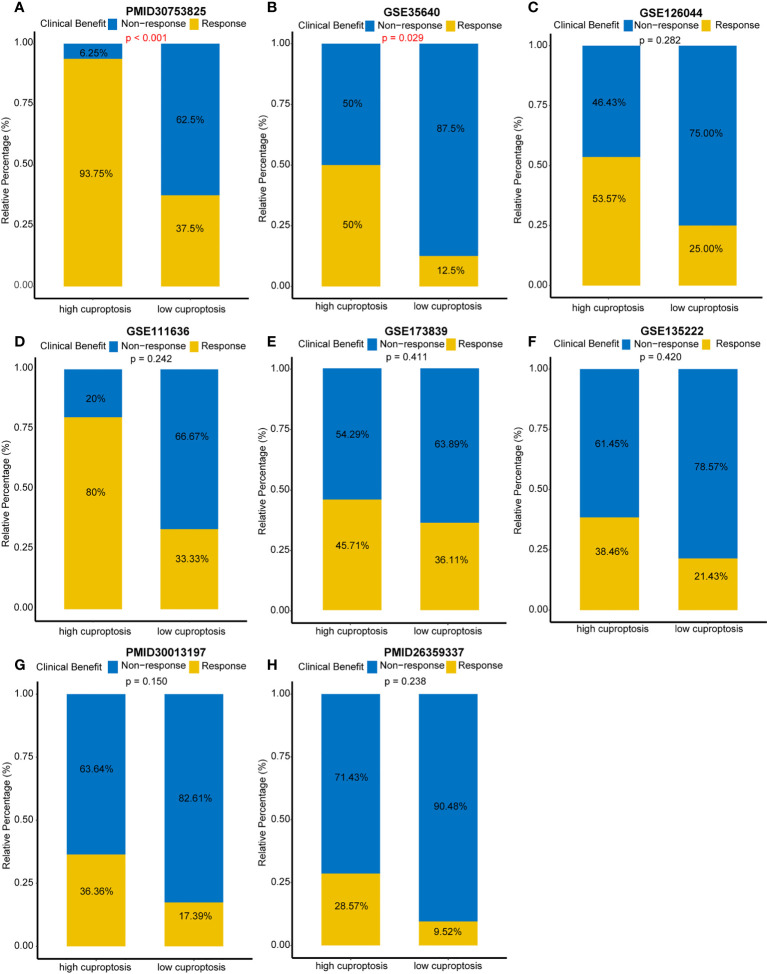
Direct comparison of immune checkpoint blockades’ efficacy in multiple immunotherapy cohorts. **(A)** Response rates between different cuproptosis signature groups in the PMID30753825 cohort. **(B)** Response rates between different cuproptosis signature groups in the GSE35640 cohort. **(C–H)** Response rates between different cuproptosis signature groups in the GSE126044, GSE111636, GSE173839, GSE135222, PMID30013197, and PMID26359337 cohorts, respectively.

Despite the fact that we evaluated an individual’s efficacy of ICB by cuproptosis signature, it is necessary to directly compare the curative effect of ICB cohorts in the high- and low-score-signature group. Hence, eight immunotherapy cohorts were included in our study. In the PMID30753825 cohort, a 93.75% response rate occurred in the high-score group compared to 37.5% in the low-score group (*p* < 0.001) (**Figure 5A**). In the GSE35640 cohort, the response rate ratio was 50 *versus* 12.5% between two groups (*p* = 0.029) (**Figure 5B**). Although there were no significant differences between the two groups in the other six cohorts (GSE124044, GSE111636, GSE173839, GSE135222, PMID30013197, and PMID26359337), patients with a high score showed a better curative effect (**Figures 5C–H**). These findings further demonstrated that cuproptosis signature is qualified to forecast the efficacy of ICB and provide guidance for the precise application of immunotherapy.

### Assessing the prognosis of individuals using cuproptosis risk score

**Figure 6:**
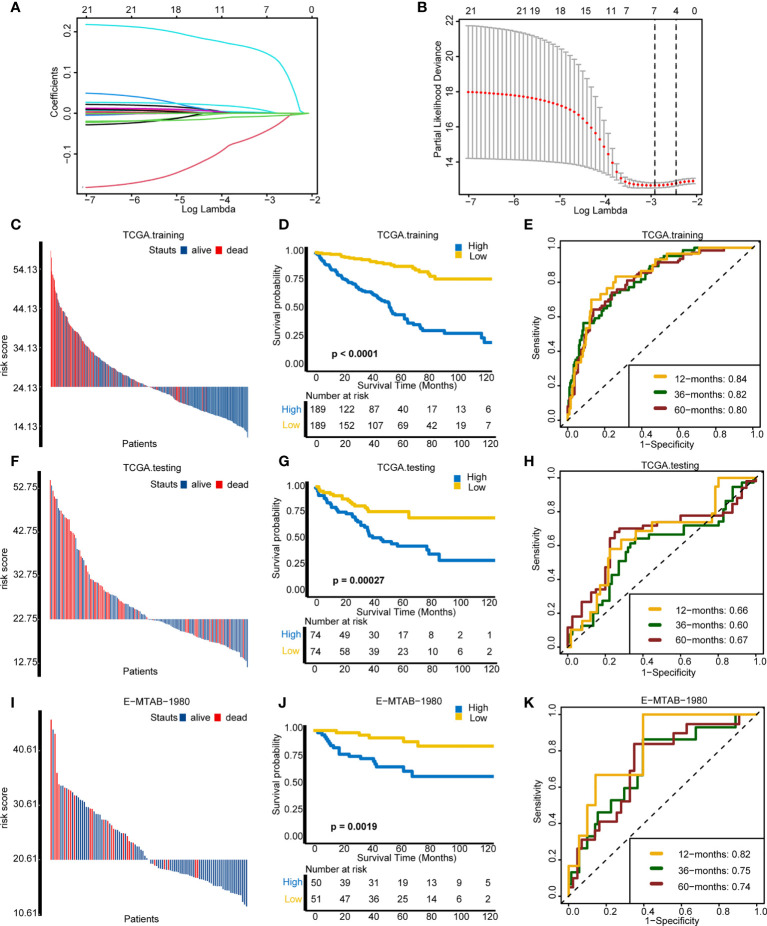
Assessing the prognosis of individuals using the cuproptosis risk score. **(A)** Coefficients of 21 cuproptosis-related genes with prognostic value. **(B)** Cross-validation of parameter selection based on the minimum criteria of LASSO regression model. **(C–E)** Comparisons of survival events, Kaplan–Meier (K–M) survival curves, and time-dependent receiver operating characteristic (ROC) curves between different risk score groups in TCGA training cohort. **(F–H)** Comparisons of survival events, (K–M) survival curves, and time-dependent ROC curves between different risk score groups in The Cancer Genome Atlas testing cohort. **(I–K)** Comparisons of survival events, (K–M) survival curves, and time-dependent ROC curves between different risk score groups in the external validation cohort (E-MTAB-1980).

Then, we developed and validated a robust cuproptosis risk score for predicting the survival outcome of an individual. LASSO algorithm was used to select the optimal candidate genes. As a result, seven candidate genes (SLC31A2, SCO2, MT2A, DBH, UBE2D3, CCL8, and MT1X) with minimal lambda (0.05) were chosen from the 43 cuproptosis-associated genes (**Figures 6A, B**). Subsequently, the cuproptosis risk score was built using the “rfsrc” function in “randomForestSRC” R package, and the risk score was significantly positively correlated with cuproptosis signature (**Supplementary Figure S5A**).

On the basis of the median value of the risk score, individuals were divided into a high-risk group and a low-risk group. In the TCGA training cohort, the high-risk group comprised more death cases and worse survival probability (*p* < 0.0001) than the low-risk group (**Figures 6C, D**). Moreover, the prediction accuracy of the survival outcomes at 12, 36, and 60 months were 0.84, 0.82, and 0.80, respectively (**Figure 6E**). In the TCGA internal validation cohort, the survival status and the survival probability (*p* = 0.00027) between the two groups were in accordance with the outcomes of the TCGA training cohort (**Figures 6F, G**). The area under the curve (AUC) of the survival outcomes at 12, 36, and 60 months were 0.66, 0.60, and 0.67, respectively (**Figure 6H**). Importantly, in the external validation cohort (E-MTAB-1980), individuals in the low-risk group manifested better survival status and survival probability (*p* = 0.0019) in comparison with the high-risk group (**Figures 6I, J**). The prediction accuracy of prognosis at 12, 36, and 60 months were 0.82, 0.75, and 0.74, respectively (**Figure 6K**). In line with the outcome that patients with a high cuproptosis risk score exhibited a worse OS, patients with higher tumor grade and stage had a significantly higher risk score (**Supplementary Figures S5B, C**). However, there were no differences on risk score between different ages and genders (**Supplementary Figures S5D, E**). All these results demonstrated that cuproptosis risk score can precisely foretell the prognosis of a KIRC individual.

### Development of a nomogram for better forecasting survival outcome in clinical practice

**Figure 7:**
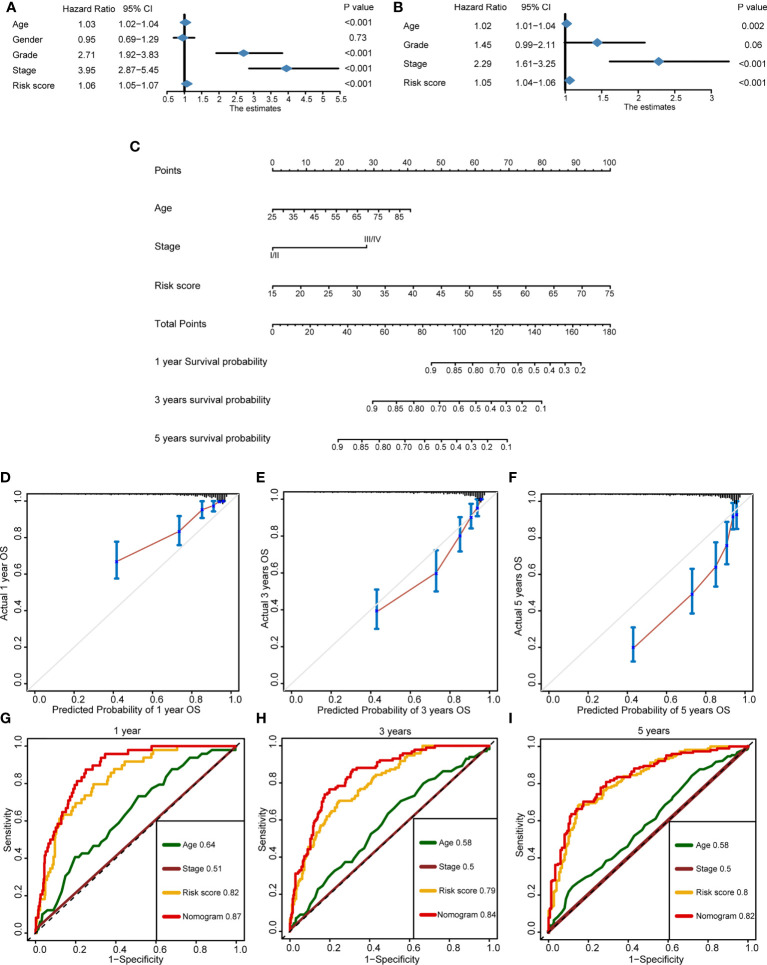
Development of a nomogram for better forecasting survival outcome in clinical practice. **(A)** Prognostic factors selected by univariate Cox regression. **(B)** Independent prognostic factors selected by multivariate Cox regression. **(C)** The nomogram-predicted overall survival at 1, 3, and 5 years by incorporating independent prognostic factors. **(D–F)** The calibration curves exhibited the clinical relevance of a nomogram at 1, 3, and 5 years. **(G–I)** Time-dependent receiver operating characteristics showed the prediction accuracy of a nomogram, risk score, age, and stage at 1, 3, and 5 years.

In order to improve the prediction accuracy of OS in clinical practice, we developed a nomogram incorporating the cuproptosis risk score and the essential clinical characteristics. In the TCGA-KIRC cohort, univariate Cox regression was used to select the prognostic factors. Except for gender (*p* = 0.73), other indicators had a significant prognostic value (*p* < 0.001) (**Figure 7A**). Then, multivariate Cox regression was applied to identify the independent prognostic factors. Cuproptosis risk score (*p* < 0.001), age (*p* = 0.002), and tumor stage (*p* < 0.001) were eligible (**Figure 7B**) and incorporated into a nomogram (**Figure 7C**). By means of calibration curves, the predicted probability values of OS at 1 year (**Figure 7D**), 3 years (**Figure 7E**), and 5 years (**Figure 7F**) were similar with the actual probability OS, which demonstrated that the nomogram has a crucial clinical value. Additionally, we compared the prediction accuracy between nomogram, cuproptosis risk score, age, and stage, respectively. The outcome indicated that the nomogram is the most precise tool to predict OS at 1 year (AUC = 0.87), 3 years (AUC = 0.84), and 5 years (AUC = 0.82) (**Figures 7G–I**).

## Discussion

Copper is the essential element for cell proliferation and cell death. Meanwhile, it is also the necessary cofactor for enzymes and transporters (39). Dysfunction of copper metabolism will cause a cytotoxic effect and an oxidative stress response in various types of cells (40, 41). Hence, we defined copper-dependent cell proliferation as cuproplasia. In contrast, copper-dependent cell death is defined as cuproptosis, whose mechanism probably is to increase the energy metabolism of the mitochondria and the accumulation of ROS (42). More importantly, recently, cuproptosis was found to have a close connection with tumorigenesis, progression, and metastasis. Li et al. demonstrated that the activated cuproptosis-associated axis (IL-17-STEAP4-XIAP) can turn colon inflammation into cancer (43). Petris et al. revealed that silenced ATP7A can inhibit the progression and metastasis of lung cancer *via* altering the activity of LOX family’s enzymes (44). Furthermore, a close correlation between cuproptosis and infiltration of immune cells has been found in several studies. Paredes et al. reported that the mutation of MAP2K1 can change the abundance of macrophage, mature dendritic cell, regulatory T cell, and cytotoxic lymphocyte (45). Tan et al. revealed that ceruloplasmin plays an important role in the immune infiltration of breast cancer (46).

Based on the abundance and location of cytotoxic lymphocytes in tumor tissue and invasive margin, the TME can be sorted into inflamed (hot) type and non-inflamed (cold) types (47). It is generally thought that the type of the TME has an important influence on the efficacy of immunotherapy (48, 49)—for example, van der Burg et al. found that a higher infiltration level of CD4^+^T cells and CD8^+^T cells shows longer overall survival and recurrence-free survival in patients who accepted anti-PD-1/PD-L1 immunotherapy (50). Rabadan et al. found that converting the non-inflamed TME of glioblastoma into inflamed TME can significantly improve the response rate to anti-PD-1 therapy (51). On the foundation of a set of vital genes and unsupervised clustering, there are many studies that correlated gene patterns with prognosis and the TME phenotypes. According to the expression of 21 m6A regulators, Zhang et al. correlated the m6A modification patterns with prognosis and the characteristics of the TME cell infiltration in gastric cancer (52). Chen et al. depicted the leukocyte infiltration level in pancreatic ductal adenocarcinoma using hypoxia- and immune-related patterns (53). Liu et al. made use of three m5C modified patterns for assessing the TME and prognosis of patients with lung adenocarcinoma (54). Based on 46 TNF-related genes, two distinct clusters were identified and employed to evaluate the immune characteristics of head and neck squamous cell carcinoma (55). For KIRC, two m6A-related patterns were found and used to predict the immune phenotypes and the efficacy of immunotherapy in our previous study (56). However, as far as we are concerned, this is the first study that systematically correlates cuproptosis-related genes with the immune infiltration level and prognosis in KIRC. We found that cuproptosis-related patterns are able to distinguish the subtypes of TME and survival outcome well. In addition, for evaluating the immune infiltration characteristic on an individual, ssGSEA algorithm was used to construct a cuproptosis signature, which is qualified to predict the inflamed level of the TME on an individual. Furthermore, multiple immunotherapy cohorts were employed to directly compare the response rate between high- and low-cuproptosis-signature groups. These findings are crucial indicators for supplying the precise therapy in KIRC.

More importantly, to predict the survival performance on an individual, cuproptosis risk score was built and showed accurate prediction in testing and multiple validating cohorts. Han et al. developed a cuproptosis-associated lncRNA risk score for prognosis and TME phenotype prediction in soft tissue sarcoma (57). However, their risk score was only validated in the TCGA internal cohort. For KIRC, Xu et al. developed a glycolysis-related risk score and correlated it with prognosis and the TME characteristics. Although they found that their risk score was an independent risk factor, its predictive accuracy in the validation cohort was unclear (58). Similar to Xu’s study, Chen et al. and Xing et al. developed and validated a necroptosis and an autophagy-related risk score on the basis of the 11 related genes (59, 60). Our risk score built based on seven cuproptosis-related genes was more convenient for clinical application than Xing’s 11 autophagy-related genes. While for Chen’s study, though their risk score was successfully validated in the TCGA internal validation cohort, the predictive accuracy still needs further study. Different from most of the risk scores developed using Cox proportional hazard regression analysis, random survival forest (RSF) was used to construct a cuproptosis risk score, with satisfied reliability and extrapolation. In our previous study, precision and robustness of cox proportional hazard regression and RSF have been compared. The outcome indicated that RSF shows a better performance (61). Similarly, Yang et al. built prediction model by six different algorithms and found that RSF was optimal solution with best accuracy (62). It is considered that the excellent multi-process control property of RSF is the key. To sum up, cuproptosis risk scores were constructed by LASSO regression and RSF and validated in multiple cohorts for the first time. It is valuable for predicting the prognosis and conducting a precision treatment for a patient.

Inevitably, there are some limitations in the study. First of all, the follow-up time of the Xiangya-RCC cohort was not enough, which led to it being unqualified as an external validation cohort for prognosis. Secondly, both the training cohort and the validation cohort were retrospective cohorts; it is necessary to further validate the risk score in prospective cohorts. Thirdly, the underlying mechanism of cuproptosis in the TME still needs to be explored *in vitro* and *in vivo*.

## Conclusion

Distinct cuproptosis-related patterns have significant differences on prognosis and immune cell infiltration in KIRC. Cuproptosis signature and risk score are able to provide guidance for precision therapy and accurate prognosis prediction for patients with KIRC.

## Data availability statement

The datasets presented in this study can be found in online repositories. The names of the repository/repositories and accession number(s) can be found in the article/**Supplementary Material**.

## Ethics statement

This study was reviewed and approved by Medical Ethics Committee of Xiangya Hospital Central South University (approval number:202104145). Written informed consent was obtained from all participants for their participation in this study.

## Author contributions

Conception and design: ZC, HL, ZL, and XZ. Collection and assembly of data: ZC and ZY. Statistical analyses: HL, JH, and ZX. Manuscript writing: ZC, HL, and YH. All authors contributed to the article and approved the submitted version.

## Funding

This study was supported by the National Natural Science Foundation of China (81873626, and 82070785) and Hunan Province Key R&D Program (2019SK2202).

## Acknowledgments

We sincerely thank all participants in the study.

## Conflict of interest

The authors declare that the research was conducted in the absence of any commercial or financial relationships that could be construed as a potential conflict of interest.

## Publisher’s note

All claims expressed in this article are solely those of the authors and do not necessarily represent those of their affiliated organizations, or those of the publisher, the editors and the reviewers. Any product that may be evaluated in this article, or claim that may be made by its manufacturer, is not guaranteed or endorsed by the publisher.

## Supplementary material

Supplementary Figure 1 Kaplan–Meier survival curves of 21 cuproptosis-related genes with prognostic value.

Supplementary Figure 2 Consensus score matrix of the TCGA-KIRC samples when set *k* was 2, 3, 4, and 5 separately.

Supplementary Figure 3 Detailed cell markers in recognized cell types. **(A)** Cancer cell. **(B)** Endothelial cells. **(C)** Vascular smooth muscle cell. **(D)** Macrophage cell. **(E)** T cell. **(F)** B cell. **(G)** Mast cell.

Supplementary Figure 4
**(A)** Expression patterns of cuproptosis-related genes in cancer cells and non-cancer cells. **(B)** Heat map of differentially expressed genes (DEGs) between cuproptosis-related patterns. **(C)** Volcano plot of DEGs between two patterns; several immune-related genes were marked. **p* < 0.05, ***p* < 0.01, ****p* < 0.001; ns, not statistically significant.

Supplementary Figure 5
**(A)** Correlation between cuproptosis signature and risk score. **(B**, **C)** Cuproptosis risk score between different tumor grades and stages. **(D**, **E)** Cuproptosis risk score between different ages and genders.

Supplementary Table 5 Gene Ontology enrichment pathway analysis based on differentially expressed genes between two patterns.

Supplementary Table 6 Kyoto Encyclopedia of Genes and Genomes enrichment pathway analysis based on differentially expressed genes between two patterns.

## Glossary

**Table:**

KIRC	kidney renal clear cell carcinoma
TME	tumor microenvironment
ICB	immune checkpoint inhibitors
OS	overall survival
RCC	renal cell carcinoma
ROS	reactive oxygen species
CTR-1	copper transporter 1
PD-1	programmed cell death 1
PD-L1	programmed cell death 1 ligand 1
CTLA-4	cytotoxic T lymphocyte-associated antigen-4
MAGE-A3	Melanoma antigen family A-3
TCGA	The Cancer Genome Atlas
DEGs	differentially expressed genes
FC	fold change
GSEA	gene set enrichment analysis
NES	normalized enrichment score
GO	Gene Ontology
KEGG	Kyoto Encyclopedia of Genes and Genomes
TIP	Tracking Tumor Immunophenotype
ssGSEA	single-sample gene set enrichment analysis
TIS	T cell inflamed score
LASSO	least absolute shrinkage and selection operator
K–M	Kaplan–Meier
ROC	receiver operating characteristic
AUC	area under curve
TILs	tumor infiltrating leukocytes
MCP	microenvironment cell populations
Quantiseq	quantification of the tumor immune contexture from human RNA-seq data
TIMER	tumor immune estimation resource
NK cell	nature killer
Th1 cell	type 1 T helper cell
scRNA-seq	single-cell RNA sequencing
VSM	vascular smooth muscle
m6A	N6-methyladenosine
TNF	tumor necrosis factor
RSF	random survival forest

## References

[1] Ljungberg B Albiges L Abu-Ghanem Y Bensalah K Dabestani S Fernández-Pello S. European Association of urology guidelines on renal cell carcinoma: The 2019 update. Eur Urol (2019) 75(5):799–810. doi: 10.1016/j.eururo.2019.02.011 30803729

[2] Siegel RL Miller KD Fuchs HE Jemal A. Cancer statistics, 2021. CA: Cancer J Clin (2021) 71(1):7–33. doi: 10.3322/caac.21654 33433946

[3] Simonaggio A Epaillard N Pobel C Moreira M Oudard S Vano YA. Tumor microenvironment features as predictive biomarkers of response to immune checkpoint inhibitors (ICI) in metastatic clear cell renal cell carcinoma (mccRCC). Cancers (2021) 13(2):231–53. doi: 10.3390/cancers13020231 PMC7827724 33435262

[4] Larroquette M Peyraud F Domblides C Lefort F Bernhard JC Ravaud A. Adjuvant therapy in renal cell carcinoma: Current knowledges and future perspectives. Cancer Treat Rev (2021) 97:102207. doi: 10.1016/j.ctrv.2021.102207 33906023

[5] Motzer RJ Tannir NM McDermott DF Arén Frontera O Melichar B Choueiri TK. Nivolumab plus ipilimumab versus sunitinib in advanced renal-cell carcinoma. N Engl J Med (2018) 378(14):1277–90. doi: 10.1056/NEJMoa1712126 PMC5972549 29562145

[6] Braun DA Bakouny Z Hirsch L Flippot R Van Allen EM Wu CJ. Beyond conventional immune-checkpoint inhibition - novel immunotherapies for renal cell carcinoma. Nat Rev Clin Oncol (2021) 18(4):199–214. doi: 10.1038/s41571-020-00455-z 33437048 PMC8317018

[7] Duan Q Zhang H Zheng J Zhang L. Turning cold into hot: Firing up the tumor microenvironment. Trends Cancer (2020) 6(7):605–18. doi: 10.1016/j.trecan.2020.02.022 32610070

[8] Gajewski TF. The next hurdle in cancer immunotherapy: Overcoming the non-T-Cell-Inflamed tumor microenvironment. Semin Oncol (2015) 42(4):663–71. doi: 10.1053/j.seminoncol.2015.05.011 PMC4555998 26320069

[9] Havel JJ Chowell D Chan TA. The evolving landscape of biomarkers for checkpoint inhibitor immunotherapy. Nat Rev Cancer (2019) 19(3):133–50. doi: 10.1038/s41568-019-0116-x PMC6705396 30755690

[10] Riaz N Havel JJ Makarov V Desrichard A Urba WJ Sims JS. Tumor and microenvironment evolution during immunotherapy with nivolumab. Cell (2017) 171(4):934–49.e16. doi: 10.1016/j.cell.2017.09.028 29033130 PMC5685550

[11] Topalian SL Drake CG Pardoll DM. Immune checkpoint blockade: a common denominator approach to cancer therapy. Cancer Cell (2015) 27(4):450–61. doi: 10.1016/j.ccell.2015.03.001 PMC4400238 25858804

[12] Tumeh PC Harview CL Yearley JH Shintaku IP Taylor EJ Robert L. PD-1 blockade induces responses by inhibiting adaptive immune resistance. Nature (2014) 515(7528):568–71. doi: 10.1038/nature13954 PMC4246418 25428505

[13] Ge EJ Bush AI Casini A Cobine PA Cross JR DeNicola GM. Connecting copper and cancer: From transition metal signalling to metalloplasia. Nat Rev Cancer (2022) 22(2):102–13. doi: 10.1038/s41568-021-00417-2 PMC8810673 34764459

[14] Blockhuys S Wittung-Stafshede P. Roles of copper-binding proteins in breast cancer. Int J Mol Sci (2017) 18:871–81. doi: 10.3390/ijms18040871 PMC5412452 28425924

[15] Kardos J Héja L Simon Á Jablonkai I Kovács R Jemnitz K. Copper signalling: causes and consequences. Cell Commun Signaling CCS (2018) 16(1):71. doi: 10.1186/s12964-018-0277-3 30348177 PMC6198518

[16] Voli F Valli E Lerra L Kimpton K Saletta F Giorgi FM. Intratumoral copper modulates PD-L1 expression and influences tumor immune evasion. Cancer Res (2020) 80(19):4129–44. doi: 10.1158/0008-5472.Can-20-0471 32816860

[17] Goldman MJ Craft B Hastie M Repečka K McDade F Kamath A. Visualizing and interpreting cancer genomics data *via* the xena platform. Nat Biotechnol (2020) 38(6):675–8. doi: 10.1038/s41587-020-0546-8 PMC7386072 32444850

[18] Guo T Duan H Chen J Liu J Othmane B Hu J. N6-methyladenosine writer gene ZC3H13 predicts immune phenotype and therapeutic opportunities in kidney renal clear cell carcinoma. Front Oncol (2021) 11:718644. doi: 10.3389/fonc.2021.718644 34497769 PMC8420859

[19] Gide TN Quek C Menzies AM Tasker AT Shang P Holst J. Distinct immune cell populations define response to anti-PD-1 monotherapy and anti-PD-1/Anti-CTLA-4 combined therapy. Cancer Cell (2019) 35(2):238–55.e6. doi: 10.1016/j.ccell.2019.01.003 30753825

[20] Kim ST Cristescu R Bass AJ Kim KM Odegaard JI Kim K. Comprehensive molecular characterization of clinical responses to PD-1 inhibition in metastatic gastric cancer. Nat Med (2018) 24(9):1449–58. doi: 10.1038/s41591-018-0101-z 30013197

[21] Van Allen EM Miao D Schilling B Shukla SA Blank C Zimmer L. Genomic correlates of response to CTLA-4 blockade in metastatic melanoma. Sci (New York NY) (2015) 350(6257):207–11. doi: 10.1126/science.aad0095 PMC5054517 26359337

[22] Wilkerson MD Hayes DN. ConsensusClusterPlus: A class discovery tool with confidence assessments and item tracking. Bioinf (Oxford England) (2010) 26(12):1572–3. doi: 10.1093/bioinformatics/btq170 PMC2881355 20427518

[23] Xu L Deng C Pang B Zhang X Liu W Liao G. TIP: A web server for resolving tumor immunophenotype profiling. Cancer Res (2018) 78(23):6575–80. doi: 10.1158/0008-5472.Can-18-0689 30154154

[24] Charoentong P Finotello F Angelova M Mayer C Efremova M Rieder D. Pan-cancer immunogenomic analyses reveal genotype-immunophenotype relationships and predictors of response to checkpoint blockade. Cell Rep (2017) 18(1):248–62. doi: 10.1016/j.celrep.2016.12.019 28052254

[25] Hu J Yu A Othmane B Qiu D Li H Li C. Siglec15 shapes a non-inflamed tumor microenvironment and predicts the molecular subtype in bladder cancer. Theranostics (2021) 11(7):3089–108. doi: 10.7150/thno.53649 PMC7847675 33537076

[26] Auslander N Zhang G Lee JS Frederick DT Miao B Moll T. Robust prediction of response to immune checkpoint blockade therapy in metastatic melanoma. Nat Med (2018) 24(10):1545–9. doi: 10.1038/s41591-018-0157-9 PMC6693632 30127394

[27] Zhang Y Narayanan SP Mannan R Raskind G Wang X Vats P. Single-cell analyses of renal cell cancers reveal insights into tumor microenvironment, cell of origin, and therapy response. Proc Natl Acad Sci USA (2021) 118(24):e2103240118. doi: 10.1073/pnas.2103240118 34099557 PMC8214680

[28] Hu J Chen Z Bao L Zhou L Hou Y Liu L. Single-cell transcriptome analysis reveals intratumoral heterogeneity in ccRCC, which results in different clinical outcomes. Mol Ther J Am Soc Gene Ther (2020) 28(7):1658–72. doi: 10.1016/j.ymthe.2020.04.023 PMC7335756 32396851

[29] Friedman DJ Crotts SB Shapiro MJ Rajcula M McCue S Liu X. ST8Sia6 promotes tumor growth in mice by inhibiting immune responses. Cancer Immunol Res (2021) 9(8):952–66. doi: 10.1158/2326-6066.Cir-20-0834 PMC8338779 34074677

[30] Lin X Wu JF Wang DM Zhang J Zhang WJ Xue G. The correlation and role analysis of KCNK2/4/5/15 in human papillary thyroid carcinoma microenvironment. J Cancer (2020) 11(17):5162–76. doi: 10.7150/jca.45604 PMC7378911 32742463

[31] Chen DS Mellman I. Oncology meets immunology: The cancer-immunity cycle. Immunity (2013) 39(1):1–10. doi: 10.1016/j.immuni.2013.07.012 23890059

[32] Chen DS Mellman I. Elements of cancer immunity and the cancer-immune set point. Nature (2017) 541(7637):321–30. doi: 10.1038/nature21349 28102259

[33] Giraldo NA Becht E Remark R Damotte D Sautès-Fridman C Fridman WH. The immune contexture of primary and metastatic human tumours. Curr Opin Immunol (2014) 27:8–15. doi: 10.1016/j.coi.2014.01.001 24487185

[34] Liu Z Han C Fu YX. Targeting innate sensing in the tumor microenvironment to improve immunotherapy. Cell Mol Immunol (2020) 17(1):13–26. doi: 10.1038/s41423-019-0341-y 31844141 PMC6952427

[35] Gajewski TF Corrales L Williams J Horton B Sivan A Spranger S. Cancer immunotherapy targets based on understanding the T cell-inflamed versus non-T cell-inflamed tumor microenvironment. Adv Exp Med Biol (2017) 1036:19–31. doi: 10.1007/978-3-319-67577-0_2 29275462 PMC6693322

[36] Ji RR Chasalow SD Wang L Hamid O Schmidt H Cogswell J. An immune-active tumor microenvironment favors clinical response to ipilimumab. Cancer Immunol Immunother CII (2012) 61(7):1019–31. doi: 10.1007/s00262-011-1172-6 PMC11028506 22146893

[37] Owonikoko TK Dwivedi B Chen Z Zhang C Barwick B Ernani V. YAP1 expression in SCLC defines a distinct subtype with T-cell-Inflamed phenotype. J Thorac Oncol Off Publ Int Assoc Study Lung Cancer (2021) 16(3):464–76. doi: 10.1016/j.jtho.2020.11.006 PMC7920957 33248321

[38] Romero JM Grünwald B Jang GH Bavi PP Jhaveri A Masoomian M. A four-chemokine signature is associated with a T-cell-Inflamed phenotype in primary and metastatic pancreatic cancer. Clin Cancer Res Off J Am Assoc Cancer Res (2020) 26(8):1997–2010. doi: 10.1158/1078-0432.Ccr-19-2803 31964786

[39] Michalczyk K Cymbaluk-Płoska A. The role of zinc and copper in gynecological malignancies. Nutrients (2020) 12(12):3732–53. doi: 10.3390/nu12123732 PMC7761859 33287452

[40] Que EL Domaille DW Chang CJ. Metals in neurobiology: Probing their chemistry and biology with molecular imaging. Chem Rev (2008) 108(5):1517–49. doi: 10.1021/cr078203u 18426241

[41] Shanbhag VC Gudekar N Jasmer K Papageorgiou C Singh K Petris MJ. Copper metabolism as a unique vulnerability in cancer. Biochim Biophys Acta Mol Cell Res (2021) 1868(2):118893. doi: 10.1016/j.bbamcr.2020.118893 33091507 PMC7779655

[42] Tsvetkov P Coy S Petrova B Dreishpoon M Verma A Abdusamad M. Copper induces cell death by targeting lipoylated TCA cycle proteins. Sci (New York NY) (2022) 375(6586):1254–61. doi: 10.1126/science.abf0529 PMC9273333 35298263

[43] Liao Y Zhao J Bulek K Tang F Chen X Cai G. Inflammation mobilizes copper metabolism to promote colon tumorigenesis *via* an IL-17-STEAP4-XIAP axis. Nat Commun (2020) 11(1):900. doi: 10.1038/s41467-020-14698-y 32060280 PMC7021685

[44] Shanbhag V Jasmer-McDonald K Zhu S Martin AL Gudekar N Khan A. ATP7A delivers copper to the lysyl oxidase family of enzymes and promotes tumorigenesis and metastasis. Proc Natl Acad Sci United States America (2019) 116(14):6836–41. doi: 10.1073/pnas.1817473116 PMC6452744 30890638

[45] Paredes SEY Almeida LY Trevisan GL Polanco XBJ Silveira HA Vilela Silva E. Immunohistochemical characterization of immune cell infiltration in paediatric and adult langerhans cell histiocytosis. Scandinavian J Immunol (2020) 92(6):e12950. doi: 10.1111/sji.12950 32738155

[46] Chen F Han B Meng Y Han Y Liu B Zhang B. Ceruloplasmin correlates with immune infiltration and serves as a prognostic biomarker in breast cancer. Aging (2021) 13(16):20438–67. doi: 10.18632/aging.203427 PMC8436892 34413268

[47] Galon J Bruni D. Approaches to treat immune hot, altered and cold tumours with combination immunotherapies. Nat Rev Drug Discovery (2019) 18(3):197–218. doi: 10.1038/s41573-018-0007-y 30610226

[48] Meurette O Mehlen P. Notch signaling in the tumor microenvironment. Cancer Cell (2018) 34(4):536–48. doi: 10.1016/j.ccell.2018.07.009 30146333

[49] Zemek RM De Jong E Chin WL Schuster IS Fear VS Casey TH. Sensitization to immune checkpoint blockade through activation of a STAT1/NK axis in the tumor microenvironment. Sci Trans Med (2019) 11(501):14–27. doi: 10.1126/scitranslmed.aav7816 31316010

[50] Kortekaas KE Santegoets SJ Tas L Ehsan I Charoentong P van Doorn HC. Primary vulvar squamous cell carcinomas with high T cell infiltration and active immune signaling are potential candidates for neoadjuvant PD-1/PD-L1 immunotherapy. J Immunother Cancer (2021) 9:e003671. doi: 10.1136/jitc-2021-003671 34716208 PMC8559240

[51] Zhao J Chen AX Gartrell RD Silverman AM Aparicio L Chu T. Immune and genomic correlates of response to anti-PD-1 immunotherapy in glioblastoma. Nat Med (2019) 25(3):462–9. doi: 10.1038/s41591-019-0349-y PMC6810613 30742119

[52] Zhang B Wu Q Li B Wang D Wang L Zhou YL. m(6)A regulator-mediated methylation modification patterns and tumor microenvironment infiltration characterization in gastric cancer. Mol Cancer (2020) 19(1):53. doi: 10.1186/s12943-020-01170-0 32164750 PMC7066851

[53] Chen D Huang H Zang L Gao W Zhu H Yu X. Development and verification of the hypoxia- and immune-associated prognostic signature for pancreatic ductal adenocarcinoma. Front Immunol (2021) 12:728062. doi: 10.3389/fimmu.2021.728062 34691034 PMC8526937

[54] Liu T Hu X Lin C Shi X He Y Zhang J. 5-methylcytosine RNA methylation regulators affect prognosis and tumor microenvironment in lung adenocarcinoma. Ann Trans Med (2022) 10(5):259. doi: 10.21037/atm-22-500 PMC8987885 35402591

[55] Long Q Huang C Meng Q Peng J Yao F Du D. TNF patterns and tumor microenvironment characterization in head and neck squamous cell carcinoma. Front Immunol (2021) 12:754818. doi: 10.3389/fimmu.2021.754818 34691075 PMC8526904

[56] Li H Hu J Yu A Othmane B Guo T Liu J. RNA Modification of N6-methyladenosine predicts immune phenotypes and therapeutic opportunities in kidney renal clear cell carcinoma. Front Oncol (2021) 11:642159. doi: 10.3389/fonc.2021.642159 33816290 PMC8013979

[57] Han J Hu Y Liu S Jiang J Wang H. A newly established cuproptosis-associated long non-coding RNA signature for predicting prognosis and indicating immune microenvironment features in soft tissue sarcoma. J Oncol (2022) 2022:8489387. doi: 10.1155/2022/8489387 35847354 PMC9279026

[58] Xu F Guan Y Xue L Huang S Gao K Yang Z. The effect of a novel glycolysis-related gene signature on progression, prognosis and immune microenvironment of renal cell carcinoma. BMC Cancer (2020) 20(1):1207. doi: 10.1186/s12885-020-07702-7 33287763 PMC7720455

[59] Xing Q Ji C Zhu B Cong R Wang Y. Identification of small molecule drugs and development of a novel autophagy-related prognostic signature for kidney renal clear cell carcinoma. Cancer Med (2020) 9(19):7034–51. doi: 10.1002/cam4.3367 PMC7541166 32780567

[60] Chen W Lin W Wu L Xu A Liu C Huang P. A novel prognostic predictor of immune microenvironment and therapeutic response in kidney renal clear cell carcinoma based on necroptosis-related gene signature. Int J Med Sci (2022) 19(2):377–92. doi: 10.7150/ijms.69060 PMC8795799 35165523

[61] Li H Liu S Li C Xiao Z Hu J Zhao C. TNF family-based signature predicts prognosis, tumor microenvironment, and molecular subtypes in bladder carcinoma. Front Cell Dev Biol (2021) 9:800967. doi: 10.3389/fcell.2021.800967 35174161 PMC8842074

[62] Yang L Wu H Jin X Zheng P Hu S Xu X. Study of cardiovascular disease prediction model based on random forest in eastern China. Sci Rep (2020) 10(1):5245. doi: 10.1038/s41598-020-62133-5 32251324 PMC7090086

