# Supplementary figures and images for: Cuproptosis-related modification patterns depict the tumor microenvironment, precision immunotherapy, and prognosis of kidney renal clear cell carcinoma
Source: Front Immunol. 2022 Sep 23;13:933241. doi: 10.3389/fimmu.2022.933241 (PMC9540508; doi:10.3389/fimmu.2022.933241)

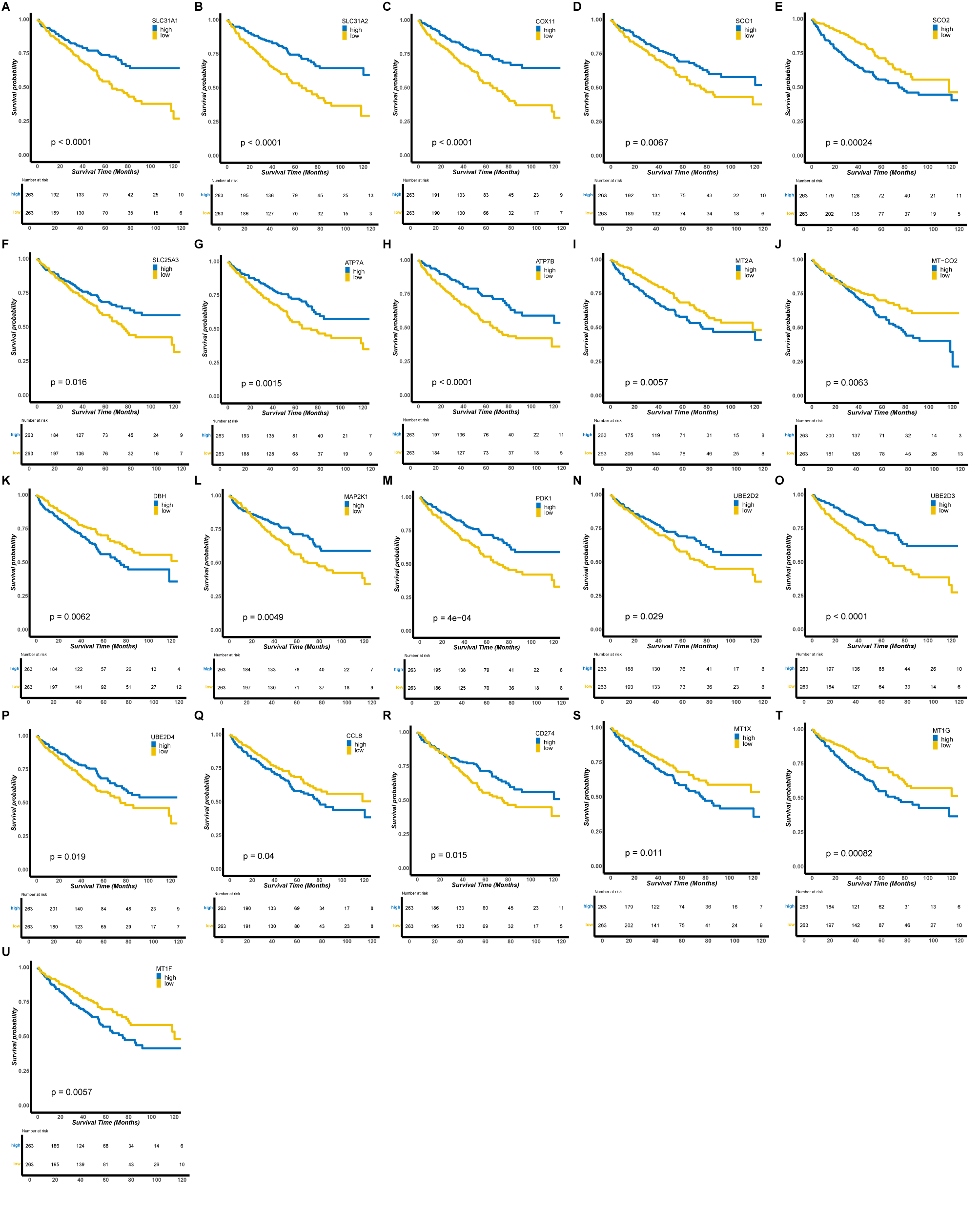

Supplement: Supplementary Figure 1 — Kaplan–Meier survival curves of 21 cuproptosis-related genes with prognostic value. [file Image_1.tif]

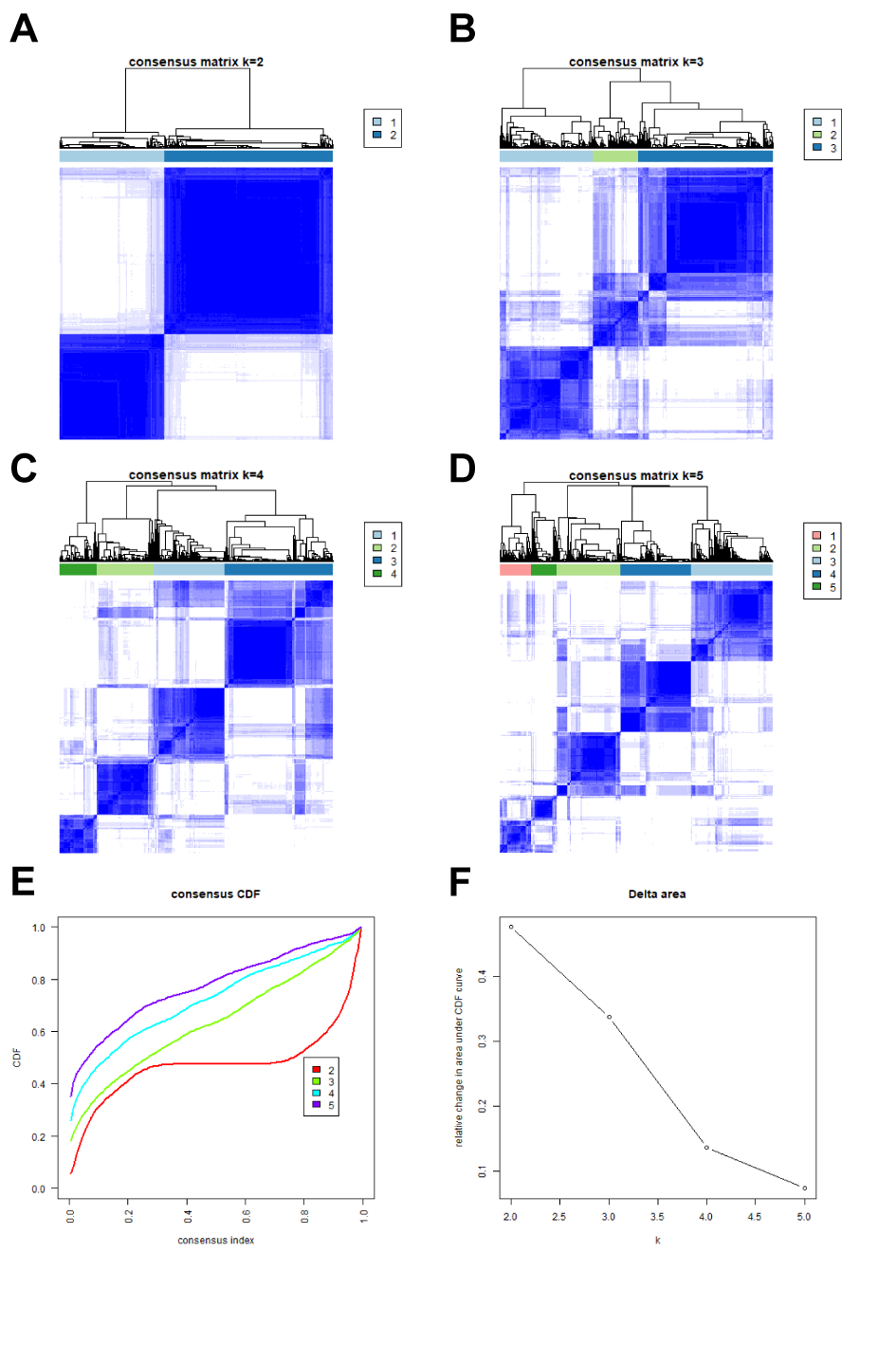

Supplement: Supplementary Figure 2 — Consensus score matrix of the TCGA-KIRC samples when set k was 2, 3, 4, and 5 separately. [file Image_2.tif]

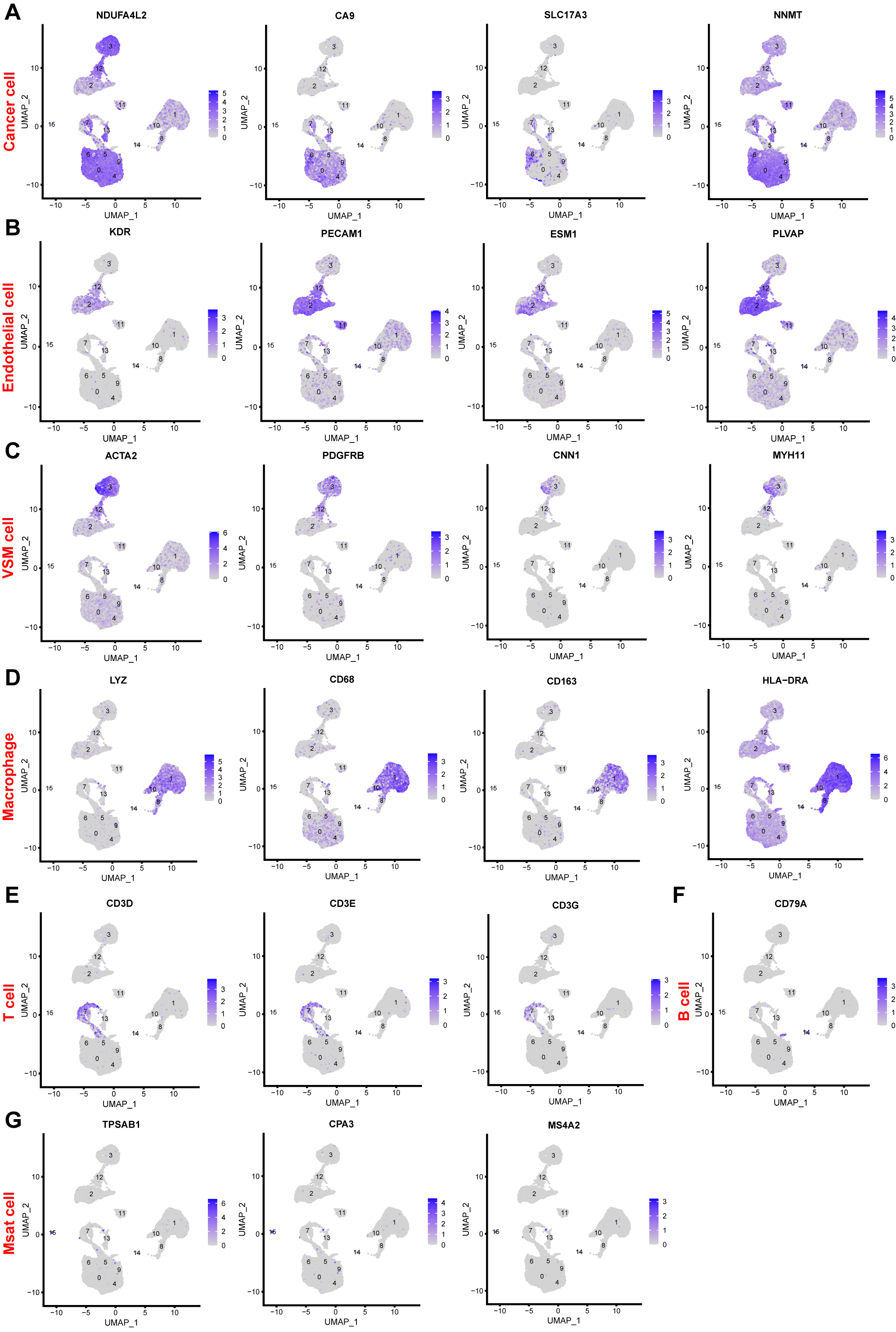

Supplement: Supplementary Figure 3 — Detailed cell markers in recognized cell types. (A) Cancer cell. (B) Endothelial cells. (C) Vascular smooth muscle cell. (D) Macrophage cell. (E) T cell. (F) B cell. (G) Mast cell. [file Image_3.tif]

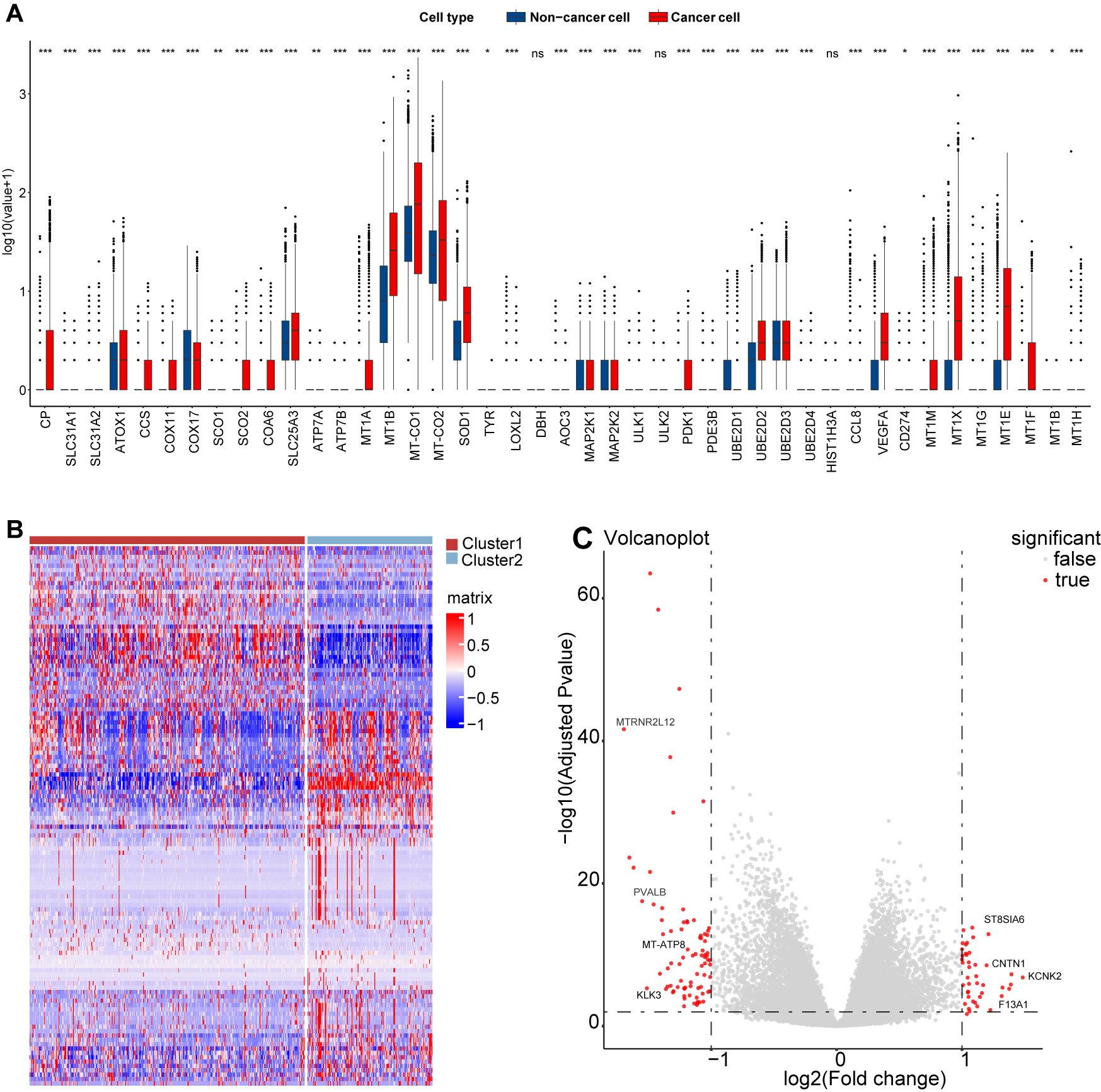

Supplement: Supplementary Figure 4 — (A) Expression patterns of cuproptosis-related genes in cancer cells and non-cancer cells. (B) Heat map of differentially expressed genes (DEGs) between cuproptosis-related patterns. (C) Volcano plot of DEGs between two patterns; several immune-related genes were marked. *p < 0.05, **p < 0.01, ***p < 0.001; ns, not statistically significant. [file Image_4.tif]

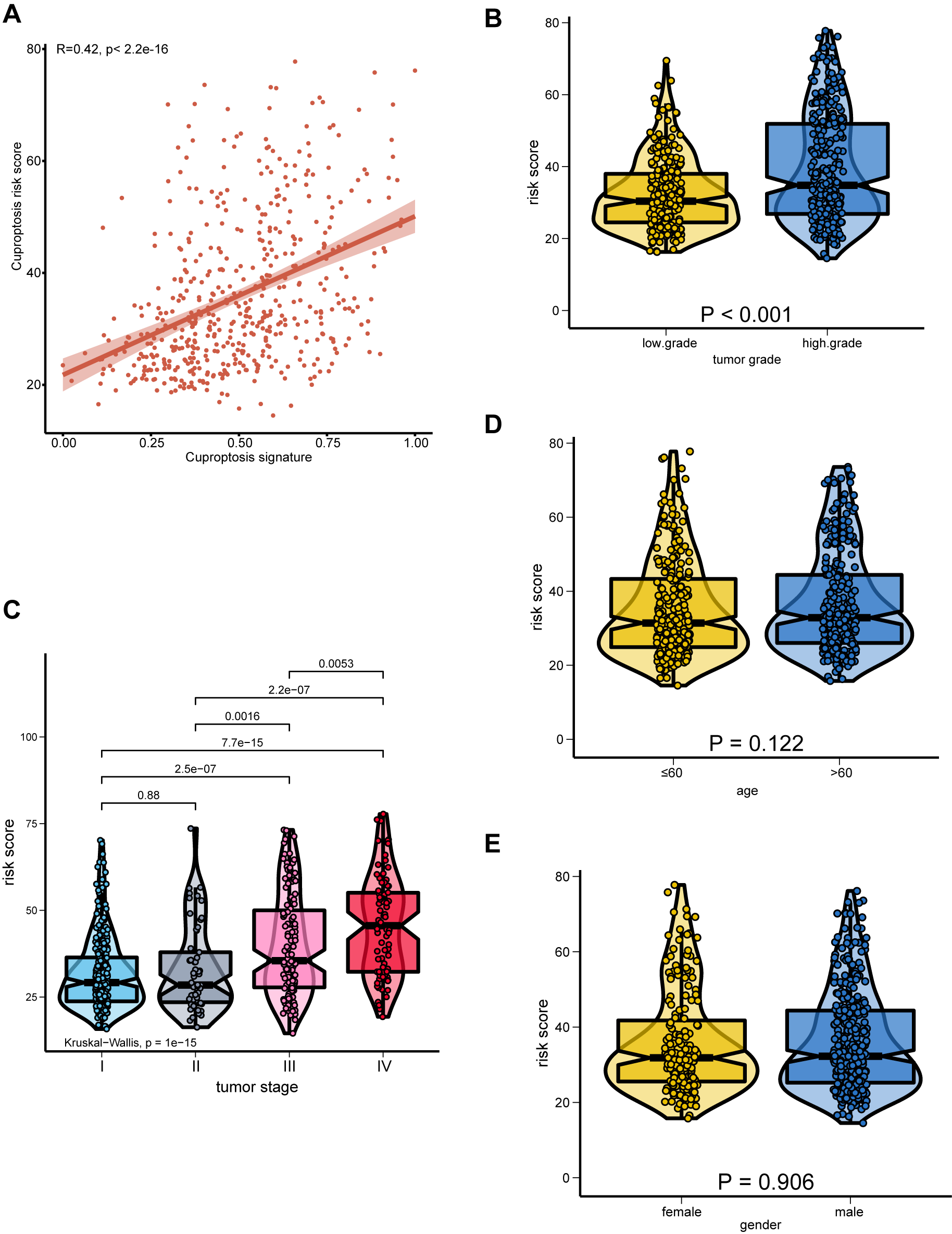

Supplement: Supplementary Figure 5 — (A) Correlation between cuproptosis signature and risk score. (B, C) Cuproptosis risk score between different tumor grades and stages. (D, E) Cuproptosis risk score between different ages and genders. [file Image_5.tif]
